# A Patient-Held Smartcard With a Unique Identifier and an mHealth Platform to Improve the Availability of Prenatal Test Results in Rural Nigeria: Demonstration Study

**DOI:** 10.2196/jmir.8716

**Published:** 2018-01-15

**Authors:** Semiu Olatunde Gbadamosi, Chuka Eze, John Olajide Olawepo, Juliet Iwelunmor, Daniel F Sarpong, Amaka Grace Ogidi, Dina Patel, John Okpanachi Oko, Chima Onoka, Echezona Edozie Ezeanolue

**Affiliations:** ^1^ Global Health Initiative School of Community Health Sciences University of Nevada, Las Vegas Las Vegas, NV United States; ^2^ Vitira Health Arlington, VA United States; ^3^ Caritas Nigeria Abuja Nigeria; ^4^ Department of Behavioral Science and Health Education St Louis University St Louis, MO United States; ^5^ Center for Minority Health and Health Disparities Research and Education Xavier University New Orleans, LA United States; ^6^ University of Nigeria Nsukka Nigeria

**Keywords:** mHealth, prenatal screening, HIV, hepatitis B, sickle cell disease, Nigeria, telemedicine, prenatal diagnosis, infectious disease transmission, vertical

## Abstract

**Background:**

Community-based strategies to test for HIV, hepatitis B virus (HBV), and sickle cell disease (SCD) have expanded opportunities to increase the proportion of pregnant women who are aware of their diagnosis. In order to use this information to implement evidence-based interventions, these results have to be available to skilled health providers at the point of delivery. Most electronic health platforms are dependent on the availability of reliable Internet connectivity and, thus, have limited use in many rural and resource-limited settings.

**Objective:**

Here we describe our work on the development and deployment of an integrated mHealth platform that is able to capture medical information, including test results, and encrypt it into a patient-held smartcard that can be read at the point of delivery without the need for an Internet connection.

**Methods:**

We engaged a team of implementation scientists, public health experts, and information technology specialists in a requirement-gathering process to inform the design of a prototype for a platform that uses smartcard technology, database deployment, and mobile phone app development. Key design decisions focused on usability, scalability, and security.

**Results:**

We successfully designed an integrated mHealth platform and deployed it in 4 health facilities across Benue State, Nigeria. We developed the Vitira Health platform to store test results of HIV, HBV, and SCD in a database, and securely encrypt the results on a Quick Response code embedded on a smartcard. We used a mobile app to read the contents on the smartcard without the need for Internet connectivity.

**Conclusions:**

Our findings indicate that it is possible to develop a patient-held smartcard and an mHealth platform that contains vital health information that can be read at the point of delivery using a mobile phone-based app without an Internet connection.

**Trial Registration:**

ClinicalTrials.gov NCT03027258; https://clinicaltrials.gov/ct2/show/NCT03027258 (Archived by WebCite at http://www.webcitation.org/6owR2D0kE)

## Introduction

### Background

Despite significant investment and efforts to address maternal and child health challenges in Nigeria, the country still has one of the highest rates of child mortality in the world, with 108 deaths among children less than 5 years of age per 1000 live births [1]. An estimated 240,000 of the 750,000 children who die before their fifth birthday are newborns. Most of these infant deaths are attributable to preventable infectious diseases, including HIV and hepatitis B virus (HBV) infections, and complications of sickle cell disease (SCD)—the commonest genetic disease [1,2]. Even though programs to prevent mother-to-child transmission (MTCT) of HIV in Nigeria have been expanded, an estimated 41,000 infants became infected with HIV in 2015 [3]. The risk of perinatal transmission of HIV is increased when an HIV-infected pregnant woman is co-infected with HBV, which remains endemic in Nigeria [4,5]. Further, children with SCD are at increased risk of HIV infection due to frequent blood transfusion [6]. In Nigeria, about 50% to 80% of children with SCD die before their fifth birthday due to several complications from the disease [7,8]. Despite the availability of simple, inexpensive interventions to prevent MTCT of HIV and HBV and to manage SCD, including antiretroviral prophylaxis, HBV vaccine, and penicillin prophylaxis, respectively, implementation remains inconsistent as a result of limited availability of diagnostic information at the point of delivery. It is therefore critical not only that pregnant women are screened for these conditions, but also that there is an efficient and effective way of ensuring these results are available at the point of delivery.

In our previous study, we had demonstrated the feasibility of using a community-based, integrated approach to increase uptake of screening for conditions such as HIV infection, HBV infection, and sickle cell genotype among pregnant women in southeastern Nigeria [9,10]. Our Healthy Beginning Initiative (HBI) trial was a cluster-randomized trial designed to reduce barriers to screening for these diseases during the prenatal period. We conducted the HBI trial among pregnant women and their male partners in communities where they resided. The scale-up of HBI in communities across Nigeria has expanded opportunities to increase the proportion of pregnant women who are tested and aware of their diagnosis. In order to use this information to implement evidence-based interventions for the prevention of MTCT of HIV and HBV and management of the infant with SCD, these test results need to be available to a skilled birth attendant at the point of delivery.

Medical record keeping is an arduous task in resource-limited settings. Efforts to implement electronic medical record (EMR) systems to improve collection and availability of and access to personal health information have been met with challenges. Caritas Nigeria, a US President’s Emergency Plan for AIDS Relief (PEPFAR)-supported implementing partner providing HIV services in Nigeria, runs an EMR system anchored in the IQCare package managed by the Palladium Group (formerly Futures Group). IQCare is deployed in locations in Nigeria, Uganda, Zimbabwe, and Kenya [11]. It is a robust and customizable database with an SQL back end and an Internet Explorer-based form, query, and report front end. It was to be configured and customized to suit either a paperless or a paper-based setup. The paperless operation mode of IQCare is the ideal setup where computers or other data collection devices are present at each data collection point and are all linked to a network server where the database resides. Electronic versions of the data collection forms are made available on these remote computers or devices such that data are captured seamlessly as part of the business process and uploaded directly into the central server database. For instance, when a patient enrolls at the medical record unit, the data are automatically available at the clinician’s office. A prescription made by the clinician can be accessed by the pharmacy in real time. However, limited infrastructure, such as poor Internet connectivity and unreliable electric power, make the paper-based mode more prevalent in this setting. The current practice is for data to be collected on structured paper-based forms and subsequently entered at one central data entry location on-site. This process creates an inefficient system whereby the information may not be easily retrievable and readily available, particularly when needed in an emergency situation such as at the point of delivery.

Specific mHealth solutions targeted at addressing this gap may offer promising opportunities to improve the provision of health services. Recent advancements in mHealth have the potential to strengthen the provision of health services, particularly in resource-limited settings. These advancements include the widespread use of mobile phones and growth in coverage of mobile cellular networks; the rapid rise in the development of mHealth apps and wearable devices; and the availability of higher data transmission speeds around the globe. Despite these advancements, low- and middle-income countries still face major challenges with implementing cost-effective, culturally acceptable, and sustainable mHealth solutions that can be integrated into health systems [12]. The use of low-cost, widely accessible mobile technologies offers an opportunity to augment the effort to digitize record keeping and ensure that vital test results are available to clinicians at the point of care.

### Objective

In this paper, we describe our work on the development and deployment of an integrated mHealth platform that is able to capture vital health information, including test results for HIV, HBV, and SCD, and encrypt it into a patient-held smartcard that can be read at the point of delivery without the need for an Internet connection.

Our rationale for focusing on HIV, HBV, and SCD for mHealth is based on several factors: (1) we found high prevalences of HIV, hepatitis B surface antigen, and sickle cell trait of 2% [10], 5%, and 22% [13], respectively, in pregnant women in the communities where HBI was implemented; (2) an integrated approach to screen for multiple diseases rather than a single condition was widely acceptable by the community; and (3) the integration of both infectious and chronic diseases highlights the double burden facing sub-Saharan Africa.

## Methods

### Ethical Consideration

This study was approved by the Institutional Review Board of the University of Nevada, Las Vegas, NV, USA, and the Nigerian National Health Research Ethics Committee. This study was registered with ClinicalTrails.gov (ClinicalTrials.gov identifier NCT03027258).

### Study Design and Settings

The study design details have been previously described [14]. We conducted the study in Benue State, north-central Nigeria. In 2012, Benue State was estimated to have a total population of 5,138,531, of whom 49.6% were female [15]. Its population is predominantly rural, and most are farmers [16]. According to the Nigeria Demographic and Health Survey, only 60% of pregnant women received prenatal care from a skilled provider for the most recent birth [17] and less than half of all deliveries in the state were attended by a skilled birth attendant [15]. Preliminary data from our ongoing US National Institutes of Health-funded study (grant no. R01HD075050; multiple principal investigators: EEE, CO) demonstrate that in Benue State, prevalence rates of HIV, HBV, and sickle cell trait among pregnant women are 7.8%, 11.1%, and 19.1%, respectively. Pregnant women are not routinely screened for HBV infection and sickle cell genotype during prenatal care in Benue State.

### Study Procedure

#### Predevelopment

In February 2016, we assembled an interdisciplinary group of experts to identify the core needs of the platform given the current challenges identified in the provision of health services in the settings. Expert consultations were conducted by (1) implementation science researchers in the United States with extensive work experience in Nigeria, (2) public health consultants, (3) computer scientists and programmers, and (4) a cadre of health workers with HIV programmatic experience based in Nigeria. The group met biweekly via videoconference during the initial stages to review evidence and share experience in concept and design to inform the development of the platform. Our objective was to develop a decentralized medical health records platform that allowed any authorized health professional to, within seconds, retrieve vital personalized health information needed to improve health outcomes. In addition, we sought to develop a solution that could provide patients with more control over their medical health record. We were also constrained by additional requirements. Our solution needed to be low cost and potentially work on any Android-based mobile phone without the need for an external Internet connection or additional physical hardware. On the basis of our constraints, we arrived at some core design and architectural decisions. Our key design decisions focused on usability, scalability, and security. Every aspect of our solution had to be capable of being deployed to new users without extensive training. Knowledge of how to use a basic mobile phone would be sufficient for a health official to download the mobile app, launch the app, scan a patient card, and use the data to effectively manage a patient. At various points during the development of both the mobile app and the back-end database, we obtained feedback from our research teams and users on the design, usability, and effectiveness of our solution.

#### Development

The mobile app and back-end database were built using various mobile and Web app software development tools. They include the JavaScript jQuery version 3.2.1 and PHP programming languages, Ionic Mobile Development Framework version 3.6.1 (Ionic), Android Software Development Kit version 26.0.1 (Google Inc), Apple Developer Tools Xcode version 7.3.1 (Apple Inc), and MariaDB version 10.2 (MariaDB Foundation) for secure database management. All mobile and Web-based back-end app code was written from scratch and was not preconfigured using an existing open-source app platform. We estimate that 96% of our mobile app code was portable between Android and iOS devices form factors (mobile phone and tablet). On the back-end database server, we developed and deployed a virtual private cloud in the Amazon Web Services (Amazon Web Services, Inc) cloud environment to securely host the back-end app. We integrated Quick Response (QR) code technology as our primary data transport medium. We quickly focused on developing our first minimum viable product. The first working prototype achieved the ability for an authorized medical health official to use the Vitira Health app to identify a patient’s medical health information within 20 seconds without the need for an external data connection and EMR solution. We followed a continuous development approach for the development of the rest of the platform. Understanding the importance of patient privacy, we developed and incorporated an algorithm to encrypt patient data on the smartcard and added user authentication capabilities into the mobile app. We put a tremendous amount of effort into the development of the Vitira Health back-end database where patient data were securely managed. When a patient card was printed, the data were encrypted, encoded, and printed onto a smartcard. We also developed the ability to pull, encrypt, and encode patient data from third-party partner EMR systems through secure application programming interfaces. We built both the mobile app and the Web back-end database to be fully scalable and deployable across multiple mobile phone device and Web architectures.

#### Predeployment

We developed a manual to help facilitate training sessions for end users and outlined defined tasks and performance objectives. A reference manual with module content delivered in screenshots and picture aids was also developed to guide users in navigating through the different menus and options of the platform. We identified and selected health facilities to deploy the Vitira Health mHealth platform based on several criteria. The facility had to (1) receive funding support from PEPFAR through Caritas Nigeria, (2) be a comprehensive treatment facility, and (3) have records of a high volume of HIV-infected patients and deliveries in the preceding year. Comprehensive treatment facility sites offer free HIV testing services, antiretroviral therapy for both adults and children, and services for prevention of MTCT.

#### Deployment

Hospital administrators at 4 health facilities (Father Matthias Hospital, Naka; Nongu u Kristu ke Sudan hen Tiv Comprehensive Health Center, Garagbohol; Nongu u Kristu ke Sudan hen Tiv Health Center, Uchi; and Mimidoo Clinic, Gungul) gave their consent to participate in the study. Health workers including birth attendants within the health facilities were selected by their respective hospital administrator to participate in a training session. SOG and JOO facilitated a 3-hour training session in each health facility. During the introductory session, participants were provided with a brief overview, key features and capabilities, and a demonstration of a prototype of the mHealth platform. Participants were then given a list of several tasks to perform to expose them to the core features of the prototype. Throughout the session, we obtained and documented valuable informal feedback from the participants and later adjusted our designs accordingly. At the end of the session, a reference manual was given to the health facility.

## Results

### Development and Deployment of the Integrated mHealth Platform

Between February and August 2016, we successfully developed an integrated mHealth platform—a point-of-care technology solution that incorporates a Web-based database, smartcard technology, and a mobile app. From September to October 2016, we deployed the Vitira Health platform at the 4 health facilities. The Vitira Health platform was designed specifically to function in remote locations with low Internet availability. We put security at the forefront of our design decisions. Health data had to be encrypted in storage and during transmission. We implemented those controls with encryption mechanisms on the smartcards, mobile app, and app database servers.

#### Vitira Health Web-Based Database

**Figure 1:**
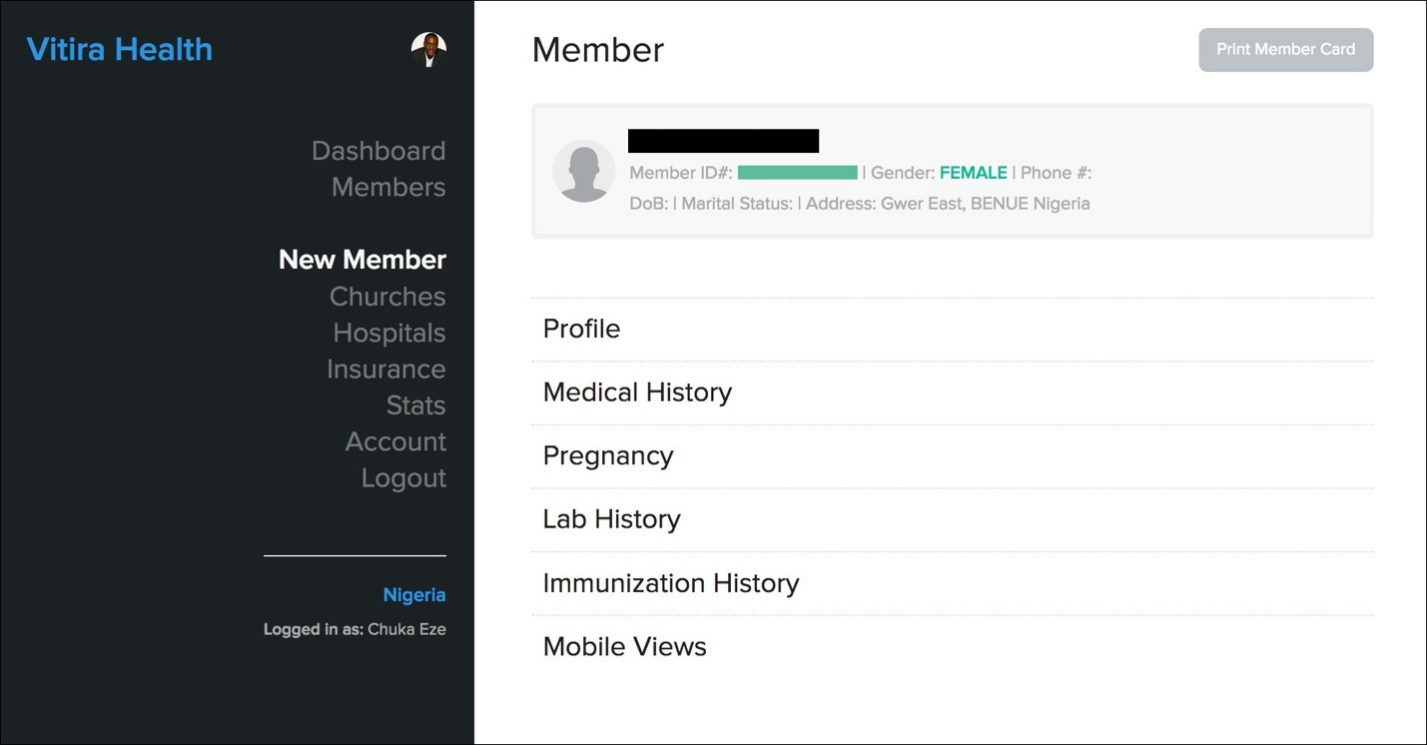
The Vitira Health Web-based database for collection of patient health information.

On the Vitira Health Web-based back-end platform, we developed the ability for authorized administrators to easily add new patient records, search patient data, and add authorized mobile app users. We also developed capabilities to perform data analytics based on information gathered from deployed apps and health records. Collected patient data, which included demographics and personal health information (Figure 1), were intelligently stored based on unique project categories such as location, disease condition, and additional features that allowed patient records to be pulled into specific slots or buckets for grouping.

Authorized skilled birth attendants (nurses and midwives) at participating health facilities were given access to the Web-based database. They were trained and assigned log-in credentials to access the platform. All access and changes to data were monitored and logged at the back end. On the basis of data collected, we could also see when and where patient records were scanned. The mobile app was able to capture date, time, and global positioning system location of patient interactions, along with the purpose of the health visit. Integration with Google Maps (Google Inc) allowed authorized management to view and track patient visits on the back end.

Multilevel access permissions allowed senior management to limit access to specific data based on permissions granted to each local user on the system. All data were backed up and managed on secure servers, at secure hosting facilities.

**Figure 2:**
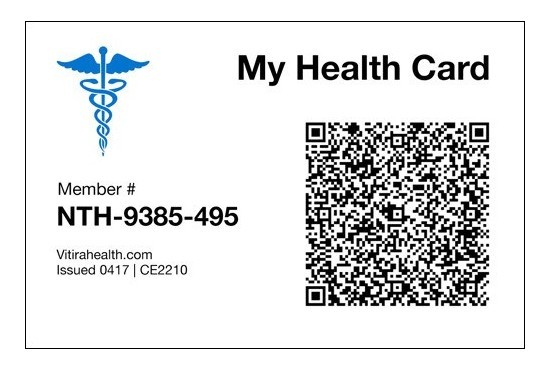
The patient-held smartcard.

#### Patient-Held Smartcard

Specific patient data, including HIV test result, date of HIV test, HBV test result, and sickle cell genotype result, were converted to an encrypted QR code and embedded on a card (Figure 2). Each smartcard had a unique identifier and was offered to pregnant women. The data encrypted in the QR code were retrieved using a mobile app to scan the smartcard.

#### Vitira Health Mobile App

**Figure 3:**
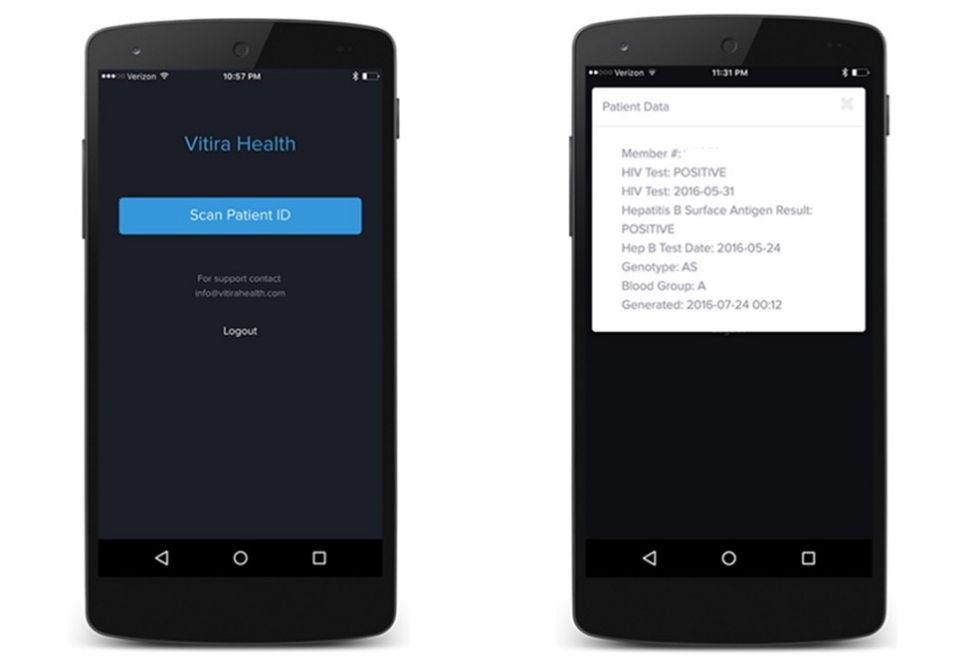
The mobile app.

The mobile app was designed as a cross-platform solution to work on Android mobile devices (with a focus on low-end, less-costly Android mobile phones) (Figure 3). We focused heavily on the workflow and sought to have a minimal number of screens and buttons on the app. We developed a modern-looking user interface that was optimized for readability. We implemented the ability for an authorized health official to authenticate once and subsequently gain quick access to the app with a personal identification number code. We developed the Vitira mobile app to work on any camera-enabled mobile phone. Our initial deployment focused on Android mobile phones due to targeted audience factors. While all mobile phones can read our QR code-embedded smartcard, we identified that phones with cameras that were 6 megapixels or higher worked more effectively.

The mobile app was used by authorized skilled birth attendants to scan the smartcard and view patients’ data. In doing this, an access log was synchronized to a cloud platform. In addition to scanning a patient’s smartcard and viewing the result on the mobile app, the date, time, and location where the smartcard was scanned was synchronized to the database. This was achieved by (1) transmitting the day, time, and location in real time if there was an available Internet connection or (2) storing these data locally on the phone if there was no Internet connectivity and transmitting it later when the connection was available. The entire mHealth platform was built with security in mind such that data across the app were transmitted with 256-bit encryption.

### Demographic Characteristics of Health Workers

A total of 19 health workers participated in the training sessions on how to use the mHealth platform. Their mean age was 32.7 (standard deviation [SD] 4.4) years. Most (16/19, 84%) were female and slightly more than half were skilled birth attendants, as Table 1 shows.

**Table 1:** Demographic characteristics of the health workers using the Vitira Health app (N=19).

Characteristics		Data
Age in years, mean (SD^a^)		32.7 (4.4)
Female sex, n (%)		16 (84)
**Job description, n**		
	Birth attendant or community health extension worker	6
	Nurse or midwife	5
	Clinic director	4
	Records or monitoring and evaluation staff	2
	Other	2

^a^SD: standard deviation.

## Discussion

We were able to demonstrate that we could develop an integrated mHealth platform comprising (1) a secure, Web-based database that contained HIV, HBV, and SCD test results data, (2) a smartcard with an embedded, encrypted, unique QR code, and (3) a mobile app capable of reading the QR code and displaying prenatal test results to authorized skilled birth attendants, even in the absence of Internet connectivity.

Effective interventions provided at the time of childbirth can reduce MTCT of HIV and HBV and guide the management of SCD. These interventions have been shown to reduce the risk of MTCT of HIV to less than 2% [18-20] and have demonstrated 85% to 90% efficacy in preventing MTCT of HBV [21,22]. However, for these interventions to be implemented at the time of childbirth, skilled birth attendants need to know the mother’s HIV, HBV, and SCD status. In Nigeria, the health system infrastructure is limited, and EMRs are nonexistent or unreliable due to Internet outages, especially in remote locations [23]. mHealth systems that can provide needed prenatal test results without relying on the Internet are needed.

Nigeria is Africa’s largest mobile market with over 150 million mobile phone users and a high penetration of Internet services through mobile networks [24]. The use of mHealth is feasible and potentially can be a cost-effective intervention for improving maternal and perinatal outcomes in Nigeria. It can be used to increase access to health information and reduce turnaround times for receipt of laboratory test results by skilled birth attendants. Studies of mHealth apps to improve maternal health in low-income countries have focused primarily on using mobile phones for data collection, appointment reminders, health promotion and education, and provider-to-provider or person-to-person communication [12,13]. Specifically in Nigeria, mHealth has been used by community health workers for decision support (prenatal care decision support algorithm), health education, and data collection, as well as by clients for appointment reminders [25,26]. To our knowledge, this is the first time that a patient-held smartcard with a unique identifier and QR code specific to the patient has been used. The QR code can be scanned using a mobile phone with an app to access maternal health data, providing needed health information at the point of delivery.

### Limitations

In this study, we demonstrated that we could develop an mHealth platform to provide maternal health data using a smartcard and a mobile phone app. Yet we do not know whether patients will (1) accept the smartcard, (2) deliver at one of the health facilities with the mobile phone capable of reading the QR code, or (3) present the smartcard when they arrive at the health facility; nor do we know whether the skilled birth attendants will use this technology to obtain maternal health data. Future research is necessary to determine the acceptability and usability of the mHealth platform on the part of participants and skilled birth attendants. Because this was a demonstration study, we did not measure the impact of mHealth on health outcomes. This is an area for future research.

A limitation to the use of the Vitira Health platform would be the potential for a security breach if unauthorized users gained access to a patient’s smartcard to read the contents of the QR code. However, available QR code readers do not have these capabilities to read the contents due to the embedded security features. We tested commonly used QR code readers publicly available on the Android platform by scanning our smartcard, and all returned scrambled and unreadable data. Also, our mobile app is not available to unauthorized users. A potential breach could occur if an unauthorized user were to gain access to both the log-in credentials and the log-in code to our mobile app to scan a QR code. However, this security breach can easily be tracked because all scans by the mobile app are synchronized to the back end and are monitored by senior-level users. As with most technological solutions, an unreliable power supply may provide challenges that hinder its use. Our mobile app is installed on mobile phone devices that are powered by rechargeable batteries. Compared with the existing system that requires an uninterrupted power supply, mobile phone batteries last longer. Also, the power supply to the mobile phones can be supplemented with power banks that are more affordable than power-generating sets, which also require a constant fuel supply.

### Conclusion

We have described our findings on the development of an integrated mHealth platform that can make test results obtained through community-based strategies available at the point of delivery. If future studies demonstrate that the mHealth platform is acceptable and usable by patients and health professionals, this technology could be applied to other chronic health conditions in which previous health data are needed at the point of care to increase the quality and effectiveness of services.

## Abbreviations

EMR: electronic medical record
HBI: Healthy Beginning Initiative
HBV: hepatitis B virus
MTCT: mother-to-child transmission
PEPFAR: President’s Emergency Plan for AIDS Relief
QR: Quick Response
SCD: sickle cell disease
SD: standard deviation
